# Supplementary material for: Transmission ecology of Trypanosoma cruzi by Rhodnius prolixus (Reduviidae: Triatominae) infesting palm-tree species in the Colombian Orinoco, indicates risks to human populations
Source: PLoS Negl Trop Dis. 2024 Feb 20;18(2):e0011981. doi: 10.1371/journal.pntd.0011981 (PMC10906903; doi:10.1371/journal.pntd.0011981)
Supplement: Attachment — Submitted filename: Responses to reviewers 261223.docx [file pntd.0011981.s003.docx]

Bogotá, Colombia

December 26^th^, 2023

Helton C. Santiago, M.D., Ph.D
Academic Editor
PLOS Neglected Tropical Diseases

Esther Schnettler
Section Editor
PLOS Neglected Tropical Diseases

Dear Doctors Santiago and Schnettler,

We thank the valuable comments of the reviewers and the editors. The English was carefully revised by a native English speaker. Please see below the specific responses to the points raised by the reviewers.

Reviewer's Responses to Questions

**Reviewer #1**

| **Comments or questions** | **Responses** |
| --- | --- |
| Reviewer #1: The hypothesis is not stated in the manuscript; The materials and methods are in accordance with the objectives and obtained results; The samples are robust and the obtained results where statistically analyzed giving support to the conclusions; There are no concerns about ethical requirements. | **R/:** We thank the comment of the reviewer, the hypothesis was included as suggested. |
| Reviewer #1: The results are clear, well presented and the statistical analysis gives support to the conclusion The tables and figs are clear and in good quality | **R/:** We thank the comment of the reviewer |
| Reviewer #1: There is no specific topic for the conclusion. We recommend a specific topic listing the main results. The discussion is well contextualized based in the literature. A few suggestions were listed in the comments bellow for the conclusions | **R/:** The conclusion was added as suggested. |
| Reviewer #1: Review Plos NTD Manuscript  number: PNTD-D-23-00601 General Comments: The manuscript entitled “Variation in the transmission ecology of Trypanosoma cruzi in Rhodnius prolixus (Reduviidae: Triatominae) among palm-tree species in the Colombian Orinoco” is well presented, written, discussed. | **R/:** We thank the comment of the reviewer |
| The M & M are in accordance with the objectives and obtained results. | **R/:** We thank the comment of the reviewer |
| The English language is nice however, a few mistakes could be found in the text therefore, a review is suggested. | **R/:** The English was revised by a native English speaker as suggested. |
| In the introduction a few references are missing. There is no contextualization neither on the triatomine vectors nor in the T. cruzi. Minor comments are listed bellow. Despite the fact the association of R. prolixus with distinct palm species has been studied by several authors, this manuscript presents relevant and original information and therefore, it has merits to be recommended for publication in the PNTD | **R/:** The introduction was improved as requested. We have also included the references as suggested. |
| Specific comments: Title: I would suggest changing it for: “ Transmission ecology of Trypanosoma cruzi in (or by?) Rhodnius prolixus (Reduviidae: Triatominae) infesting palm-tree species in the Colombian Orinoco, indicates risks to human populations”. | **R/:** The title was changed as suggested. |
| Background- We suggest writing the scientific names in full, because it is the first time of the species citation | **R/:** Corrected. The full name of the palm species was placed in the first mention. |
| Author summary- Please revise the verb in the sentence “Remarkably, it was found that the 56 kissing bugs studied, feeds on wild and domestics animal…” I think the correct is “feed” | **R/:** Done |
| Introduction- In the first paragraph, the authors briefly contextualize the Chagas disease however, very little is mentioned presenting either the etiologic agent or the triatomine vectors. We recommend including some general comments and references for both: A review of the taxonomy and biology of Triatominae subspecies (Hemiptera: Reduviidae) – Parasitology Research Do the new triatomine species pose new challenges or strategies for monitoring Chagas disease? An overview from 1979-2021 – Memórias do Instituto Oswaldo Cruz Evolution, Systematics, and Biogeography of the Triatominae, Vectors of Chagas Disease - Chapter Ecology and diversity of Trypanosoma cruzi – Acta Tropica. Trypanosoma Cruzi: An Ancient and Successful Enzootic Parasite - Infectious Tropical Diseases and One Health in Latin America. | **R/:** Done. |
| Also, after contextualizing the importance of the palms for the distribution, ecology and so on we suggest including some words on the importance of R, prolixus. This would improve the introduction | **R/:** Done. |
| Materials and methods: I would suggest including the total range of the studied area and how the humans occupy the region, also the approximate number of humans living in the area, and the number of domiciliary unities. This kind of information is highly relevant for the study carried out. | **R/:** The number of dwellings is included. However, the total area occupied by these dwellings and the number of people were not calculated; therefore, they are not included. |
| I would suggest “Triatomine sampling” instead of “Triatominae sampling”. In this topic it would be important to mention the season on which the captures were carried out. The dry and the wet seasons have relevant influences on the field work results. | **R/:** Suggestion accepted. |
| Results: Line 272- “development stages” please change for “developmental stages”. | **R/:** Corrected. |
| Line 278- In the sentence “…than in the other palm species”  I think the preposition “in” should be crossed out. | **R/:** Corrected. |
| Line 280- “sampled processed” or “samples processed”? | **R/:** Corrected |
| Line 314- “Represents the nymph ages” please change for “represent the nymphal stages”. | **R/:** Corrected. |
| Discussion: Line 343- In the sentence “Therefore, although R. prolixus is able to infest all three palm 344 species, in A. butyracea they reach bigger larger populations…” I would suggest “Therefore, although R. prolixus is able to infest all three palm species, in A. butyracea it reaches larger populations” | **R**/: Suggestion accepted. |
| Line 347- In the sentence “R. prolixus achieves large population densities” please consider changing “R. prolixus achieves higher population densities “ | **R/:** Corrected. |
| Line 281- “peridomicile environments “ please change for “ peridomiciliary environments” | **R/:** Corrected. |
| Conclusion: I would suggest including a conclusion topic. The last paragraph of the discussion could be extended listing and highlighting the most relevant findings, for instance: the numerous feeding sources including human blood as one of the most important; the obtained results in a silvatic area near human dwellings distinct from previous studies; and the importance of educational programs for people living in the area with high risk of transmission, among other relevant results. | **R/:** Done. Conclusion included. |
| Legends:  Suggestion for changes: Table 1- It would be important including the vector species name. | **R/:** Done |

**Reviewer #2**

| **Comments or questions** | **Responses** |
| --- | --- |
| Reviewer #2: -Are the objectives of the study clearly articulated with a clear testable hypothesis stated? yes, they are -Is the study design appropriate to address the stated objectives? yes, it is -Is the population clearly described and appropriate for the hypothesis being tested? yes, it is -Is the sample size sufficient to ensure adequate power to address the hypothesis being tested? see comments -Were correct statistical analysis used to support conclusions? yes, they were -Are there concerns about ethical or regulatory requirements being met? As far as I understand, I have no concern in this sense (I do not belong to their country). | **R/:** We thank the comment of the reviewer |
| Reviewer #2: I have only concerns about the way molecular material was processed. | **R/:** To prevent contamination issues, the laboratory designated six specific and isolated sub-areas for processing samples**:**  1.Sample Preparation Zone: Tissue Lysis of the insects.  2. DNA Extraction Zone: Automated extraction in the Hamilton Microlab STAR  3. Premix Zone: PCR mixtures and NGS reagents.  4. DNA Zone: Add DNA and thermocycling reaction mixture  5. Electrophoresis Zone: identifying amplicons  6. Sequencing: NGS library preparation and Oxford Nanopore Sequencing. |
| Reviewer #2: My primary concern pertains to the potential for the conclusions to inadequately represent the scientific reality. | **R/:** Done. Conclusion included. |
| Reviewer #2: The text must be dramatically improved. See below: Ln 322: higher [..insect] densities. | **R/:** Corrected. |
| Ln 333-334: “that the [the presence of all] three palm species studied”. | **R/:** Corrected. |
| Ln 334: “…as source of insect population foci of R. prolixus to transmit CD to people living …” [rewrite] | **R/:** Corrected. |
| Ln 334: delete “larger”. | **R/:** Corrected. |
| Ln 337-338: replace “host” by “inhabitant of A. butyracea”. The word “host” is often used for food sources. | **R/:** Corrected. |
| Ln 357-358: The verb "represent" agrees with which subject? | **R/:** Corrected. |
| Ln 361-363: I do not see a connection between the parasite genotype and the epidemiological importance. | **R/:** Corrected. |
| Ln 377: Delete the “the” | **R/:** Removed. |
| Ln 378: “perturbation” can be replaced by “anthropogenic action”. | **R/:** Replaced. |
| Ln 379: “perturbed” = areas under anthropogenic interference". | **R/:** Replaced. |
| Ln 381: “This could explain that the main” = “This could explain why the main”. | **R/:** Replaced. |
| Ln 392: delete “about”. | **R/:** Removed. |
| Ln 397: replace “those” by “that”. | **R/:** Replaced. |
| Ln 399: “lower [insect] densities”. | **R/:** Corrected. |
| Ln 40-401: A. butyracea must be in italics. | **R/:** Corrected. |
| Ln 406: “indices” may be replaced by “indicators”. | **R/:** Replaced. |
| Ln 407: delete the “the” before “humans”. | **R/:** Removed. |
| Reviewer #2: Major concerns The study design is commendable as the author conducted a comprehensive *R. prolixius* sampling of each of the three species of palm trees. Abiotic data has been intricately linked with biotic factors of eco-epidemiological significance. Molecular and statistical techniques were effectively employed in *R. prolixius* molecular ecology. While I appreciate the study and believe its results are worthy of publication, I do have a significant concern about the unexpectedly high frequency of first to third-stage nymph blood feedings on terrestrial animals - particularly humans. Although the recent NGS-based techniques have enhanced resolution in current studies, their heightened sensitivity increases susceptibility to contaminations. Various authors had already warned about the potential for contamination at any stage of the study involving NGS, from the field to the laboratory (see <https://doi.org/10.1371/journal.pntd.0004512)>. I believe that this issue should not be treated as a taboo. I would consider excluding human feedings. This could be done by simply adding a statement in the Materials and Methods section, indicating that “Unexpected feedings, such as those in humans at nymphal developmental stages, were excluded from the analyses.”.  Unfortunately, this would require rerunning all the analyses, but this would present a more realistic perspective. The authors are required to undertake a thorough revision of the manuscript, with a particular focus on the abstract and discussion sections. In the latter, issues were identified in nearly every line. Despite these criticisms, I reiterate that this study provides valuable information within the scope of PlosNTD. | **R/:** To prevent contamination issues, the laboratory designated six specific and isolated sub-areas for processing samples**:**  1.Sample Preparation Zone: Tissue Lysis of the insects.  2. DNA Extraction Zone: Automated extraction in the Hamilton Microlab STAR  3. Premix Zone: PCR mixtures and NGS reagents.  4. DNA Zone: Add DNA and thermocycling reaction mixture  5. Electrophoresis Zone: identifying amplicons  6. Sequencing: NGS library preparation and Oxford Nanopore Sequencing.  .  Throughout the entire procedure, we incorporate controls to avoid that our results could be affected by contamination. Moreover, the significant interaction between *R. prolixus* and humans in Casanare, where the presence of *R. prolixus* and other species, known for their inclination to feed on human blood, is particularly noteworthy (Hernandez et al., 2016, Velasquez-Ortiz, 2019).  We added the following information about the controls used in the NGS process in the methods: For the controls in our Amplicon-based Next Generation Sequencing targeting the 12S rRNA gene, we employ Genomic DNA-Rat Male Biochain® as our positive control. Additionally, we utilize RT-PCR Grade Water from Invitrogen™ as our negative control, this water undergoes testing for both prokaryotic and eukaryotic genomic DNA contamination through assessment of 16S rRNA and 18S rRNA . Importantly, our negative controls do not contain amplicons or reads from human DNA. Furthermore, we employed DNA from the *R. prolixus* colony and identified reads of *Gallus gallus*, the feeding source for these insects. |
| Minor comments or concerns Title: Although frequently used in other studies, the term “transmission ecology” does not reflect the content of the study. | **R/:** Title was modified in accordance with the suggestions of reviewer 1. |
| When first mentioning the genera of palm trees, “Attalea”, “Mauritia”, and “Acrocomia” in the Abstract (ln 28 on), please use the full name, and then provide the abbreviation for brevity. | **R/:** Corrected. The full name of the palm species was placed in the first mention. |
| Ln. 29 M&M: “resulting in 105 samples” of? | **R/:** Corrected. |
| Ln 32-34: “ …analyzed using qPCR for T. cruzi detection and discrete typing units' identification. The 33 feeding preferences were determined by sequencing the 12S rRNA gene amplicon using 34 next-generation sequencing.”. This part belongs to the M&M”. | **R/:** Corrected. |
| Ln.112-115: The objective statement appears to be rather vague. | **R/:** Corrected. |
| Ln 235-246 “Population density of R. prolixus per palm species”: The initial number of 676 R. prolixus individuals collected may seem substantial; however, when these data are stratified by palm species and insect instar, they may prove to be insufficient for robust comparisons. On the other hand, the chosen statistical tests are suitable for handling non-normally distributed data. To enhance clarity, I suggest placing the stratification details in a supplementary file (consider insertirng X, SD, Max., Min) and discussing this in the “Discussion” section. | **R/:** Done. |
| Ln 262-278: “Infection index and quantification of T. cruzi”: the same for “Population density of R. prolixus per palm species”. | **R/:** Done. |
| Ln 290: there is double parenthesis “((“. | **R/:** Corrected. |
| Discussion Although it's a matter of writing style, I recommend avoiding referencing figures and tables in the discussion. Thus, the discussion flows more smoothly when referring to the literature. | **R/:** Done. Corrected. |
| Ln 406-413: Are any of these palm trees planted by humans? This discussion only makes sense if any of the three species are used for human activities of interest. This paragraph would make much more sense in the context of anthropogenic actions if any one of them is favored by the residents. | **R/:** Suggestion solved. They are wild palms that people frequently use for building homes and feeding domestic animals. |

**Reviewer #3**

| **Comments or questions** | **Responses** |
| --- | --- |
| Reviewer #3: Please describe methods taken to ensure there was no contamination during extraction and PCR - e.g. were negative controls used, were assays run in duplicate/triplicate, were results confirmed with second molecular target, etc. Additionally, what measures were taken during bug processing/extraction to prevent contamination with human DNA that would be detected during vertebrate host analysis? | **R/:** To prevent contamination issues, the laboratory designated six specific and isolated sub-areas for processing samples**:**  1.Sample Preparation Zone: Tissue Lysis of the insects.  2. DNA Extraction Zone: Automated extraction in the Hamilton Microlab STAR  3. Premix Zone: PCR mixtures and NGS reagents.  4. DNA Zone: Add DNA and thermocycling reaction mixture  5. Electrophoresis Zone: identifying amplicons  3. Sequencing: NGS library preparation and Oxford Nanopore Sequencing.  qPCR controls: We added the next text.  Positive controls in our study involve *T. cruzi* DNA obtained from culture. As for negative extraction controls, we utilized DNA from *R. prolixus* specimens sourced from a colony free of *T. cruzi* infection. Additionally, qPCR negative controls were executed using PCR Grade Water. It's important to note that all results were considered valid only if the positive and negative controls performed as expected.  NGS controls: We added the next text  For the controls in our Amplicon-based Next Generation Sequencing targeting the 12S rRNA gene, we employ Genomic DNA-Rat Male Biochain® as our positive control. Additionally, we utilize RT-PCR Grade Water from Invitrogen™ as our negative control, this water undergoes testing for both prokaryotic and eukaryotic genomic DNA contamination through assessment of 16S rRNA and 18S rRNA . Importantly, our negative controls do not contain amplicons or reads from human DNA |
| Line 151-142: It would be helpful here to briefly elaborate on the entomological indices, infestation, clustering, colonization, and density so the reader doesn't have to look up the citation just to follow your results. For example, later in the manuscript you describe density index as "number of individuals per sampled palm" - this type of detail could be included here perhaps in parentheses for all of the different indices. | **R/:** Done |
| Line 180: Please clarify if bugs selected for feeding sources analysis were only the *T.cruzi*-positive bugs? Why? And specify whether each of the pools were formed from bugs all from the same tree, or from multiple trees of the same species**.** | **R/:** We selected *T. cruzi*-positive insects to investigate the dynamics and ecology of *T. cruzi* transmission. Our focus is exclusively on studying the mammals implicated in the transmission process. |
| Line 193: State what statistical program was used for these analyses - later you mention R but not until specifically talking about creating the graphs. | **R/:**. Finally, RCircos [57] and ggplot packages [58] of R software version 4.2.2, was used to graph the feeding sources found in *R. prolixus* individuals collected in each palm species. |
| Reviewer #3: Very nice data visualization! It may be helpful to add the Orinoco region to the map (Fig 1), as this region is mentioned several times but it was unclear to this reader exactly how that region relates to the Casanare district. | **R/:** Corrected. Region added to the map (Fig 1). |
| Reviewer #3: Please add some discussion about the possibility of contamination and whether you think this is likely or unlikely to explain the high frequency of apparent human blood meals. | R/: Considering the high sensitivity of NGS techniques and previous reports of human DNA contaminants in mosquitoes [71], we recognize the growing importance of addressing potential sources of contamination. Throughout our entire procedure, we implement controls to prevent contamination, ensuring the reliability of our results. To minimize this risk, we adhere to strict laboratory protocols, consistent with those used for the molecular diagnosis of infectious agents and implement comprehensive sample handling procedures.  Moreover, the intricate relationship between *R. prolixus* and humans in Casanare is noteworthy. The coexistence of *R. prolixus* and other species, known for their tendency to feed on human blood, is particularly notable [72,73]. This phenomenon may be explained by human activities, such as agriculture, at the collection sites, where palms are in proximity to homes, facilitating contact between vectors and human hosts. |
| Please add the details of the prevalences previously reported among R. prolixus collected from palm trees in this zone, this is needed to see how your results compare (line 352). | **R/:** Corrected. |
| Reviewer #3: Line 37-38: This sentence doesn't make sense as written - Insects... had higher abundances of R. prolixus. | **R/:** Corrected. |
| Line 41-42: The us of both "all examined insects" and "R. prolixus" in this sentence makes it sound like multiple species of insects were studied, but I don't think that is correct. | **R/:** Corrected. |
| Line 78: I'm not sure what is meant by "frequent reproductive events and leaves abscission", please clarify, especially whether reproductive events refers to the plant or the bugs? | **R/:** Corrected 3. |
| Line 86-90: This sentence is confusing, please revise for clarity. | **R/:** Corrected |
| Line 291: I believe Equus asinus would more commonly be referred to as donkeys, not horses (Equus caballus). | **R/:** Corrected. |
| Line 333-334: I don't think that the term 'significant risk factor' is appropriate here, as you have not conducted an epidemiological study looking at transmission in humans relative to the presence of palms. Could be revised as "potentially pose a significant risk for the transmission". | **R/:** Corrected. |
| Additionally, the manuscript would benefit from some additional editing for grammar. | **R/:** Done. |
| Reviewer #3: This is a very nice and interesting paper reporting systematic sampling of three species of palm trees for Rhodnius prolixus, an important vector of Trypanosoma cruzi, with parasite detection and vertebrate host meal analysis. It is certainly of value to the T. cruzi transmission ecology literature. My major concern is that the T.cruzi infection prevalence and human bloodmeal prevalence are very high, so there should be some more details and discussion about how the authors ensured this was not the result of contamination. | **R/:** Considering the high sensitivity of NGS techniques and previous reports of human DNA contaminants in mosquitoes [71], we recognize the growing importance of addressing potential sources of contamination. Throughout our entire procedure, we implement controls to prevent contamination, ensuring the reliability of our results. To minimize this risk, we adhere to strict laboratory protocols, consistent with those used for the molecular diagnosis of infectious agents and implement comprehensive sample handling procedures.  Moreover, the intricate relationship between *R. prolixus* and humans in Casanare is noteworthy. The coexistence of *R. prolixus* and other species, known for their tendency to feed on human blood, is particularly notable [72,73]. This phenomenon may be explained by human activities, such as agriculture, at the collection sites, where palms are in proximity to homes, facilitating contact between vectors and human hosts. |

Best,

The authors
