# Supplementary material for: Transmission ecology of Trypanosoma cruzi by Rhodnius prolixus (Reduviidae: Triatominae) infesting palm-tree species in the Colombian Orinoco, indicates risks to human populations
Source: PLoS Negl Trop Dis. 2024 Feb 20;18(2):e0011981. doi: 10.1371/journal.pntd.0011981 (PMC10906903; doi:10.1371/journal.pntd.0011981)
Supplement: Attachment — Submitted filename: Responses to reviewers 220124.docx [file pntd.0011981.s004.docx]

Bogotá, Colombia

January 22^nd^, 2024

Helton C. Santiago, M.D., Ph.D
Academic Editor
PLOS Neglected Tropical Diseases

Esther Schnettler
Section Editor
PLOS Neglected Tropical Diseases

Dear Doctors Santiago and Schnettler,

We thank the valuable comments of the reviewers and the editors in this second round of review. We have acknowledge the possibility of contamination as suggested by Reviewer 2 and added a section in the discussion highlighting this limitation. Please see below the specific responses to the points raised by the reviewers.

Reviewer's Responses to Questions

**Reviewer #1**

| **Comments or questions** | **Responses** |
| --- | --- |
| A coma is missing… Chagas disease, affecting approximately eight million individuals in tropical regions, is primarily transmitted by vectors. | Thanks for the comment, we added the coma as suggested. |
| I would suggest changing “resides” by inhabits or infests or colonizes | Done |
| Conclusion The term “individuals” is a bit vague. I would suggest using a more precise term | Changed as suggested |
| High population densities and infection rates were observed in each examined palm tree species. I would suggest including: High population densities and infection rates of R. prolixus were observed in each examined palm tree species. | Changed as suggested |
| In the line 170 the species name is written in full. Please change it to R. prolixus and revise all manuscript. | Changed as suggested |
| Discussion Line 363- Please correct Attalea butyracea  “A. butyracea” | Changed as suggested |
| Line 477- Please  correct R. prolixus ; after a period or to initiate a paragraph please write the scientific name in full. | Changed as suggested |

**Reviewer #2**

| **Comments or questions** | **Responses** |
| --- | --- |
| The authors presented compelling arguments to refute the possibility of contamination in the laboratory. However, they failed to acknowledge the information I provided (See https: Logue et al. 2016), indicating that contamination could also occur in the field, during insect captures. This poses a significant challenge, particularly in studies involving highly sensitive methods to detect food sources.  This potential source of contamination cannot be dismissed and warrants further discussion. This is especially crucial since nymphs in their early developmental stages exhibit extremely limited mobility. This is challenging to conceive that N1 can descend from a palm tree to feed on humans and then return to the tree - considering the observed high prevalence of feeding on humans during these sessile stages. In the discussion, it is important to acknowledge and consider the possibility of contamination in the field, as highlighted, to ensure an accurate evaluation of the research findings.  See: Logue K, Keven JB, Cannon MV, Reimer L, Siba P, Walker ED, et al. (2016) Unbiased Characterization of Anopheles Mosquito Blood Meals by Targeted High-Throughput Sequencing. PLoS Negl Trop Dis 10(3): e0004512. <https://doi.org/10.1371/journal.pntd.0004512>. | We appreciate your invaluable comments. Based on your observations, we have incorporated the following information into the methods and discussion:  **Methods:**  **DNA Extraction**:  Here, we included 5 extraction controls (RT-PCR Grade Water from Invitrogen was utilized, undergoing comprehensive testing for both prokaryotic and eukaryotic genomic DNA contamination, assessed through 16S rRNA and 18S rRNA evaluations) that were all negative suggesting very low levels of laboratory contamination.  **PCR**:  Furthermore, qPCR negative controls were executed using PCR Grade Water. It is essential to highlight that all results were deemed valid only if the positive and negative controls performed as expected, ensuring the reliability of the outcomes.  **ABS for feeding sources:**  For negative control purposes, RT-PCR Grade Water from Invitrogen was utilized, undergoing comprehensive testing for both prokaryotic and eukaryotic genomic DNA contamination, assessed through 16S rRNA and 18S rRNA evaluations [55]. It is crucial to note that negative controls were void of amplicons or reads from human DNA. Additionally, DNA from the *R. prolixus* colony (only fed with *Gallus gallus* blood) was utilized to identify reads associated with *Gallus gallus*, confirming it as the feeding source for these insects.  **Discussion**  Therefore, the question of how nymphal stages, especially N1-N3, approach humans and domestic animals for feeding remains unanswered. However, various studies have identified human blood in wild nymphs of other *Rhodnius* species, such as *R. pallescens* in Panama [71] and Colombia [72], *R. ecuadoriensis* in Ecuador [73], and *R. stali* and *R. montenegrensis* in Bolivia [74]. These findings suggest that feeding behaviors, like kleptohematophagy, could shed light on the presence of food sources from domestic environments in nymphal stages with limited mobility [70]. Additionally, the intriguing relationship between *R. prolixus* and humans in Casanare deserves attention. The coexistence of *R. prolixus* with other species, known for their tendency to feed on human blood, is particularly noteworthy [72,75]. This phenomenon may be explained by human activities, such as agriculture, at the collection sites. Palms located near homes create favorable conditions for vectors to come into contact with human hosts. Principio del formulario  Given the high sensitivity of Next-Generation Sequencing (NGS) techniques and prior reports of human DNA contaminants in mosquitoes [76], we acknowledge the growing significance of addressing potential sources of contamination. Throughout our entire procedure, we have implemented stringent controls to forestall contamination, ensuring the reliability of our results. To mitigate this risk, we adhere to rigorous laboratory protocols aligned with those employed in the molecular diagnosis of infectious agents, coupled with the implementation of comprehensive sample handling procedures. In our study, we implemented rigorous measures, including the use of 5 controls during DNA extraction (which automated for decrease the risk of contamination) and 10 controls (Eukaryotic DNA-free water) during PCR for the assessment of food sources (12S). Furthermore, we incorporated 10 controls from colony-fed individuals with Gallus gallus, where no traces of human DNA were detected, ensuring the absence of contamination in the laboratory. Nevertheless, it is crucial to acknowledge that the identification of human-origin food sources in nymphal stages with limited mobility could potentially result from contamination during field collection. Therefore, the incorporation of negative controls during the collection of triatomines in future studies will be essential[76]. |
| The authors present the results they have obtained, but it's important to acknowledge that these findings may be biased. |  |
| Logue et al. (2016) mentioned "Here, we included 30 extraction (water) controls that were all negative suggesting very low levels of laboratory contamination (if any). An interesting complementary control, which would also control for field contamination, would be to analyze male mosquitoes collected at the same time." For mosquitoes, only females feed on humans. The authors could insert a similar statment, such as:  Here, we incorporated [...insert your cautions...] controls, all of which yielded negative results, indicating minimal to no laboratory contamination. A complementary control, which could additionally address potential field contamination, would involve analyzing non-hematophagous insects collected simultaneously  [modify for your convenience] |  |

| **Comments or questions** | **Responses** |
| --- | --- |
| The additions to the methods to describe positive and negative controls and the additional information provided to reviewers on the separate stations for processing are encouraging. However, I would still like to see some details on the precautions taken to prevent human contamination of samples prior to extraction - what PPE was used (gloves, sleeves, lab coat, hair net?), were extractions conducted in biosafety cabinet or benchtop, etc. | In our laboratory, sample processing is carried out following all the regulations required for handling high-risk biological samples. Considering that field insects may be infected by the parasite despite being stored in ethanol, which inactivates the parasite, all personnel involved in insect processing and handling use all protective barriers, such as gowns, gloves, caps, and masks. Additionally, samples, prior to extraction, are manipulated in a type A2 biosafety cabinet and subsequently extracted by an automated extraction system. All these procedures ensure that there is no contamination of the samples with human DNA. However, we acknowledge that the likelihood of contamination in the field by human DNA, and we have highlighted that limitation in the discussion section. |
| How were the 115 triatomines subjected to DNA extraction selected from the 676 total collected? Was this a systematic process? Random? Stratified? It does not appear that the number of bugs tested from each tree species was representative of the overall distribution of number of bugs collected. Was a power calculation or any other stats done to determine the sample size that would be tested? | We added the next information in methods.  We selected individuals for *T. cruzi* detection, ensuring representation from all stages of *R. prolixus* across various palm species. Our approach involved choosing a minimum of 10% and up to 100% of insects from each stage within every palm species, resulting in a total of 115 individuals. This selection process was based on estimating the sample size using the most recent *T. cruzi* positivity rate in *R. prolixus* individuals from palms in Casanare, which was 85.6%. With a 90% confidence level and an accepted error of 5.5%, we determined that a sample size of 115 individuals would be sufficient. Specifically, the distribution of individuals was as follows: 54% (18/33) for *M. flexuosa*, 21.2% (56/264) for *A. aculeata*, and 11% (41/379) for *A. butyracea*. |
| It's not clear that there is good statistical support for the statement in lines 395-396 and 398-401 regarding differences in triatomine infection prevalence between tree species given the sample size tested was a subset of the total insects collected and details are missing on how that subset was selected. | The sampling strategy encompassed all nymphal stages across the three palm species, ensuring a representative selection. Specifically, the distribution of individuals was as follows: 54% (18/33) for *M. flexuosa*, 21.2% (56/264) for *A. aculeata*, and 11% (41/379) for *A. butyracea*. Notably, we included a minimum of 10% representation from each stage in every specimen.  In response to valuable feedback from the reviewer, we have modified the presentation to enhance the descriptive clarity of our findings:  Concerning *T. cruzi* infection, *R. prolixus* collected in *M. flexuosa* exhibited a slightly lower percentage, while *A. butyracea* hosted insects with the maximum parasite infection rate. This aligns with previous reports for the region by Urbano et al. (85.2%) [23], Calderón et al. (28%) [34], Rendón et al. (60.2%) [37], and Velásquez-Ortiz et al. (84%) [52]. The presence of these wild palms in the zone [29] poses a direct threat Chagas transmission in rural areas. |

Best,

The authors
